# “As long as you can make it to work the next day”: normalized drinking and problem recognition among individuals in recovery from alcohol use disorder

**DOI:** 10.3389/fpsyt.2026.1981903

**Published:** 2026-10-19

**Authors:** Monique T. Cano, Oscar F. Rojas Perez, Li Yan McCurdy, Colleen Mistler, Jordan Paris, Alexis Ramirez-Hardy, Lisa M. Fucito

**Affiliations:** 1 Department of Psychiatry, Yale University School of Medicine, New Haven, CT, United States; 2 Department of Social and Behavioral Sciences, Yale University School of Public Health, New Haven, CT, United States; 3 New York University, New York, NY, United States; 4 Yale University, New Haven, CT, United States

**Keywords:** alcohol normalization, alcohol recovery, alcohol use disorder, problem recognition, problematic alcohol use, qualitative research, social norms, socioenvironmental factors

## Abstract

**Introduction:**

Problematic alcohol use contributes substantially to morbidity, mortality, and social harm, yet may remain underrecognized by those experiencing it. Social and environmental contexts strongly influence alcohol use and drinking norms. However, less is known about how these contexts relate to recognizing one’s own drinking as problematic. Therefore, this qualitative study explored how adults in recovery from alcohol use disorder (AUD) described socioenvironmental influences on the normalization, sustained use, and recognition of problematic alcohol use.

**Method:**

Fifteen adults in recovery from AUD were recruited through recovery networks, treatment settings, and community organizations. Participants completed demographic questionnaires and semi-structured qualitative interviews conducted in person or via videoconference. Data were analyzed using Braun and Clarke’s reflexive thematic analysis.

**Results:**

Five interrelated themes and subthemes were identified: (1) Social normalization and sustained problematic alcohol use, (1a) early exposure to drinking norms, (1b) continued normalization of drinking in adult life; (2) social comparison and minimization of drinking behavior; (3) alcohol-related consequences in relationships and daily life; (4) recognizing problematic drinking amid recognizable consequences; and (5) reflections on reasons for drinking.

**Conclusion:**

Findings extend socioenvironmental perspectives on alcohol use by suggesting that social and environmental contexts may influence not only drinking practices and norms, but also how individuals understand and recognize their own alcohol use as problematic. Prevention and early intervention efforts may benefit from moving beyond crisis-driven models of care and promoting recognition of problematic alcohol use before severe consequences occur. Clinical assessment may support earlier recognition by exploring how individuals define problematic drinking and the social reference points they use to evaluate their own drinking.

## 1 Introduction

Alcohol use contributes substantially to preventable morbidity and mortality worldwide (1, 2). In the United States, alcohol-related deaths increased by about 29% during 2020–2021 compared with 2016–2017 (3). Higher levels and heavier patterns of alcohol consumption can become problematic and are associated with a wide range of adverse outcomes, including chronic disease, injury, suicide risk, relationship conflict, domestic violence, and legal involvement (4–7). The economic burden associated with excessive alcohol use in the United States is substantial, with annual costs estimated at $249 billion (8). These costs are largely attributed to health care expenditures, lost productivity, and alcohol-related harms such as motor vehicle accidents (9).

The development and sustained use of problematic drinking are influenced by interactions among biological, psychological, social, cultural, and environmental factors (10, 11). Social Cognitive Theory (SCT) provides a theoretical basis for understanding alcohol use, with particular attention to social learning and environmental influences (12). According to SCT, alcohol use is influenced by reciprocal interactions among personal factors (affective, biological, and cognitive), behavior, and the environment (12–14). The social-ecological framework complements SCT by recognizing that influences on health and behavior occur across a spectrum, from the individual level to broader societal levels (15). Within this framework, individuals are embedded within immediate environments, such as the home, workplace, and school, that exist within larger community and societal contexts (15). Broader societal factors, such as media and advertising, may influence alcohol-related attitudes and norms within family and peer networks, which can influence individual behavior (16). Across both SCT and the social-ecological framework, alcohol use may be learned and reinforced through repeated exposure, social modeling, and prevailing norms within family, peer, community, and broader socioenvironmental contexts (12, 16, 17). These social and cultural norms can provide important reference points for what patterns of alcohol use are regarded as typical or acceptable (18). Local environments may reinforce these norms through the availability and visibility of alcohol (19). For example, greater alcohol availability, including higher outlet density, has been associated with increased alcohol consumption and perceptions that drinking is common and socially endorsed (19). Characteristics of alcohol retail environments, including outlet location and late-night operating hours, may further contribute to permissive drinking environments and the normalization of alcohol use (19).

The socioenvironmental contexts through which drinking is viewed as typical or acceptable may also influence whether individuals recognize their own alcohol use as problematic. Perceptions of drinking norms are context dependent, such that what is considered typical or acceptable may vary across social settings (20). The norms of close others, such as close friends, may also be more influential than those of more distant social groups (e.g., general peers) (20). These norms can provide a basis for social comparison when individuals evaluate their own drinking (21). In social environments where heavier alcohol use is common, an individual’s own drinking may appear less problematic by comparison (22). Similarly, individuals may be less likely to recognize their drinking as problematic when they perceive themselves as different from people associated with more severe or stigmatized alcohol problems (23).

Prior research has examined problem recognition primarily through individual psychological and behavioral factors, such as insight, readiness to change, identity-related resistance, help-seeking and treatment engagement, as well as significant life disruptions and recovery (23–28). Qualitative studies have also explored perceptions of problematic drinking in specific contexts including primary care, online help-seeking, and in response to alcohol health messaging (29–32). More broadly, a recent scoping review noted that relatively little is known about how individuals subjectively understand their own alcohol-related problems (33). This review also found that most qualitative studies in this area involved participants engaged in treatment (33). Despite this growing literature, and well-established evidence that social and environmental contexts influence alcohol use and drinking norms (19, 20), less is known about how these factors relate to problem recognition (34). This broader socioenvironmental perspective may be particularly relevant to alcohol use given that it is widely embedded within culturally normative practices and socially accepted settings (35). Recent work has also emphasized the need to better understand socioenvironmental influences on problem recognition, noting that qualitative inquiry in this subject area would be valuable (34). Individuals in recovery may offer an important perspective for examining how problematic alcohol use is understood and recognized. Accordingly, the primary objective of this study was to qualitatively explore how individuals in recovery from alcohol use disorder (AUD) described socioenvironmental influences on the normalization, sustained use, and recognition of problematic alcohol use.

## 2 Materials and methods

This study was situated within an interpretivist/constructivist paradigm which assumes that reality is socially constructed, and meaning is shaped through individuals’ experiences and interpretations (36). This orientation informed the study’s focus on identifying the patterns across participants’ retrospective accounts of socioenvironmental factors associated with the normalization, sustained use, and recognition of problematic alcohol use. SCT and the social-ecological framework provided conceptual background but did not guide the interview questions, coding, or theme development. The authors also followed the Journal Article Reporting Standards for Qualitative Research (JARS-Qual) guidelines for reporting qualitative methods (37).

### 2.1 Recruitment

Participants in recovery from alcohol use were recruited between June 18, 2025 and September 9, 2025 through community partnerships and study flyers. Flyers describing the study purpose, procedures, and compensation were posted at non-profit organizations, substance use recovery centers, and treatment clinics in the Northeastern United States. Community partners also shared study information within recovery networks and invited the lead author to attend recovery support meetings to introduce the study, answer questions, and engage with potential participants. Study procedures were approved by the Yale University Institutional Review Board (HIC #2000039596).

### 2.2 Participant eligibility and screening

Participants were screened for eligibility using a brief structured screener administered by the lead author. Eligibility criteria included: 1) age ≥18 years, 2) ability to complete study procedures in English or Spanish, 3) self-identification as being in recovery from AUD, 4) ability to provide informed consent, and 5) sustained remission from AUD for ≥12 months, consistent with the DSM-5-TR sustained remission specifier (38). Recovery and remission were treated as related but distinct constructs. We operationalized recovery through self-identification as being in recovery from AUD, with sustained remission from AUD for at least 12 months as an additional eligibility criterion. Self-identification as being in recovery was not defined as synonymous with abstinence, and abstinence was not required for participation.

During screening, participants were asked whether they had ever received a formal diagnosis of AUD from a treatment provider and about their treatment history. Participants were asked the duration of their remission from AUD-related symptoms. To further assess alcohol use history, the Alcohol Use Disorders Identification Test (AUDIT) (39) was administered using a retrospective framing. Participants were asked to respond to the AUDIT items with reference to the period when their alcohol use and alcohol-related problems were most severe. This approach was used to obtain a structured assessment of prior alcohol-related difficulties among individuals currently in remission. Similar screening strategies have been used in qualitative research examining alcohol use history and recovery experiences, which often combine structured symptom assessment with self-identification of prior alcohol problems (40). Prior research in treatment-seeking populations found AUDIT scores ≥7 to correspond to a diagnostic threshold for mild AUD (41); accordingly, this score was used as a screening cutoff indicating an increased likelihood of AUD.

### 2.3 Interviews

Participants who expressed interest in the study contacted the lead author to determine eligibility. Participants who met the study criteria provided informed consent either electronically or in person, depending on the interview format. Participants then completed a demographic questionnaire. Semi-structured interviews lasted approximately 50–90 minutes. Participants had the option to complete the interview in person (*n* = 10; 66.7%) or virtually via a HIPAA-compliant version of the videoconferencing platform, Zoom (42) (*n* = 5; 33.3%). A sample of 15 participants was considered adequate for an in-depth reflexive thematic analysis given the depth of the 50–90 minute interviews and the resulting qualitative dataset (43, 44). Although participants were eligible to complete study procedures in English or Spanish, all interviews were conducted in English; therefore, translation was not required. All interviews were conducted by the lead author and audio-recorded with participants’ consent. Participants received a $50 gift card upon completion of the interview.

### 2.4 Interview guide

The interview questions were informed by the literature on problematic alcohol use, AUD and AUD treatment (45). The interview protocol was refined through consultation with a community advisory committee composed of researchers, clinicians, and individuals with lived experience of AUD and recovery (46). The final interview questions explored topics such as lived experience with alcohol use, recovery from AUD, barriers and facilitators to alcohol treatment, and recommendations to improve AUD treatment. Given participants’ recovery status, questions about alcohol use elicited reflections on prior drinking experiences as well as experiences of recovery. **Table 1** displays interview questions relevant to the present analysis.

**Table 1:** Sample semi-structured interview questions.

Interview questions and probes
• How do people in your community (e.g., family, friends) talk about drinking? o *Probe*: How has this affected how you drink?
• How would you describe someone who struggles with alcohol use?
• What does it/has it looked like when you struggle with alcohol use?
• What has your experience been with alcohol treatment/recovery?
• What prompted you to start treatment/recovery?
• How ready or prepared did you feel to start treatment/engage in recovery?

### 2.5 Interviewer background

The semi-structured interviews were conducted by the lead author, a licensed bicultural and bilingual clinical research psychologist of Latino ethnic background. At the time of the study interviews, she held a faculty appointment in the Department of Psychiatry at a medical school in the Northeastern United States. Her research broadly focuses on substance use disorders and health-related interventions for co-occurring disorders. The lead author has extensive knowledge and experience recruiting and working with hard-to-research communities with substance use disorders (47–49).

### 2.6 Reflexivity statement

The lead author’s personal and professional identities influenced every step of the research project, from study design to data analysis. The entire coding team (MTC, OFRP, LYM, JP), which included the lead author, identifies as bilingual and bicultural, with three having Latino backgrounds and one having Southeast Asian origins. Three coding team members, including the lead author, are faculty members, and one is a third-year undergraduate student majoring in Global Public Health and Biology. The undergraduate coding team member had approximately two years of research mentorship and training under the lead author, including training and experience in qualitative coding, and was supervised by the lead author throughout the analytic process.

All three faculty members have extensive experience conducting research and/or providing clinical care to communities of diverse backgrounds with AUD. As a result, the coding team’s representation provided a critical lens for exploring the data. Prior to and throughout the analytical process, the coding team reflected on how their personal and professional identities influenced their perspectives on the research topic, decision-making, and approach to data exploration. During the analytic process, the coding team engaged in reflection and conversation on how being substance use scientist-practitioners and/or personal experience with alcohol can lead to unique biases in interpreting the data. This can lead to an overemphasis or reliance on professional knowledge and personal experiences rather than on diverse perspectives from participants’ lived experiences. Through the discussion process, the coding team was able to reduce the likelihood of overlooking or undervaluing the experiences of participants with whom they shared (or did not share) backgrounds. Finally, members of the coding team debriefed both before and after coding meetings to further reduce bias and manage preconceived perceptions.

### 2.7 Data analysis

The audio-recorded interviews were transcribed verbatim by a HIPAA-compliant transcription service company. Transcripts were checked for accuracy by a member of the coding team. Each transcript was independently coded by two team members and subsequently reviewed and discussed by the broader coding team. The involvement of multiple coders was intended to bring different perspectives to the interpretation of the data (50). Stigmatizing language (e.g., “alcoholics”) and profanity in the transcripts were retained to accurately convey the participants’ sentiments.

Data were analyzed using Dedoose software version 10.0.59 (51). Participant interviews were analyzed using Braun and Clarke’s (52) reflexive thematic analysis, a flexible approach that allows for detailed, nuanced description of the phenomenon under investigation. The analysis was inductive, and coding was primarily semantic to focus on the explicit content of participants’ accounts. Theme development involved interpretative consideration of patterned meaning across the data set. The analysis also took an experiential orientation, seeking to understand participants’ contextually situated experiences and interpretations of alcohol use and recovery. Following Braun and Clarke’s six-step approach (53), coding team members first familiarized themselves with the data by reading transcripts in full and documenting initial observations. Second, the team generated initial codes through inductive engagement with the data. The coding team met weekly to review and compare coded transcripts, discuss similarities and differences in interpretations, and consider alternative ways of understanding the data. Differences in coding were treated as opportunities for reflexive discussion and further interpretation. Through these discussions, codes were iteratively refined as the analysis developed. Third, the coding team generated initial themes from the codes and compiled relevant information for each theme. Fourth, the initial themes were collaboratively reviewed in relation to the coded information and the entire dataset. Team discussions were used to interrogate, refine, and deepen the developing interpretations and to consider alternative perspectives. Fifth, themes were further refined and named. In the sixth step, the coding team identified representative extracts that illustrated the themes.

## 3 Results

### 3.1 Participant characteristics

The sample consisted of 15 individuals in recovery from AUD between the ages of 26 and 82 years (M = 51.5, SD = 13.2). Seven participants (*n* = 7; 46.7%) were recruited through word-of-mouth referrals within recovery networks, four (*n* = 4; 26.7%) through email listservs within recovery networks, and four (*n* = 4; 26.7%) through study flyers. About half of the participants self-identified as men (*n* = 8; 53.3%), and the rest identified as women (*n* = 7; 46.7%). Most of the sample self-identified as White (*n* = 11; 73.3%), followed by African American/Black (*n* = 5; 33.3%), Native American (*n* = 2; 13.3%), and Asian (*n* = 1; 6.7%). Seven participants (46.7%) reported some college education without a bachelor’s degree, three (20.0%) reported some high school education, two (13.3%) reported a high school diploma or GED, two (13.3%) reported a master’s degree, and one (6.7%) reported a bachelor’s degree. Nearly half of the sample reported an annual income of less than $25,000 (*n* = 7; 46.7%), 26.7% reported no income (*n* = 4), and smaller proportions reported annual incomes of $25,000–$50,000 (*n* = 1; 6.7%), $50,000–$100,000 (*n* = 1; 6.7%), or $100,000–$200,000 (*n* = 2; 13.3%). Regarding retrospectively reported childhood social class, most participants self-identified as having grown up in either a working-class (*n* = 6; 40.0%) or lower-middle-class (*n* = 6; 40.0%) household, with the remaining participants identifying as upper-middle class (*n* = 3; 20.0%). In terms of current social class, most participants self-identified as working-class (*n* = 12; 80.0%), followed by upper-middle class (*n* = 2; 13.3%), and lower-middle class (*n* = 1; 6.7%). Participant demographics are represented in **Table 2**.

**Table 2:** Participant demographics.

Different demographic categories	*n* (%)
Gender *n* (%)	
Men	8 (53.3)
Women	7 (46.7)
Age, in years (mean, SD)	51.5 (13.2)
Race/Ethnicity *n* (%)	
Asian	1 (6.7)
African American/Black	5 (33.3)
Native American	2 (13.3)
White	11 (73.3)
Education *n* (%)	
Completed some high school	3 (20.0)
High school diploma or GED	2 (13.3)
Some college credit	7 (46.7)
Bachelor’s degree	1 (6.7)
Master’s degree	2 (13.3)
Income *n* (%)	
No income	4 (26.7)
Less than $25,000	7 (46.7)
$25,000 - $50,000	1 (6.7)
$50,000 - $100,000	1 (6.7)
$100,000 - $200,000	2 (13.3)
Childhood social class *n* (%)	
Working class	6 (40.0)
Lower middle class	6 (40.0)
Upper middle class	3 (20.0)
Current social class *n* (%)	
Working class	12 (80.0)
Lower middle class	1 (6.7)
Upper middle class	2 (13.3)
Current involvement in support groups *n* (%)	8 (53.3)

Participants were permitted to select more than one racial identity; therefore, percentages do not sum to 100%.

### 3.2 Qualitative themes

Analysis generated five interrelated themes and two subthemes (1): Social normalization and sustained problematic alcohol use, (1a) early exposure to drinking norms, (1b) continued normalization of drinking in adult life (2); social comparison and minimization of drinking behavior; (3) alcohol-related consequences in relationships and daily life; (4) recognizing problematic drinking amid recognizable consequences; and (5) reflections on reasons for drinking. **Figure 1** represents a thematic summary of the socioenvironmental contexts surrounding problematic alcohol use and its recognition.

**Figure 1:**
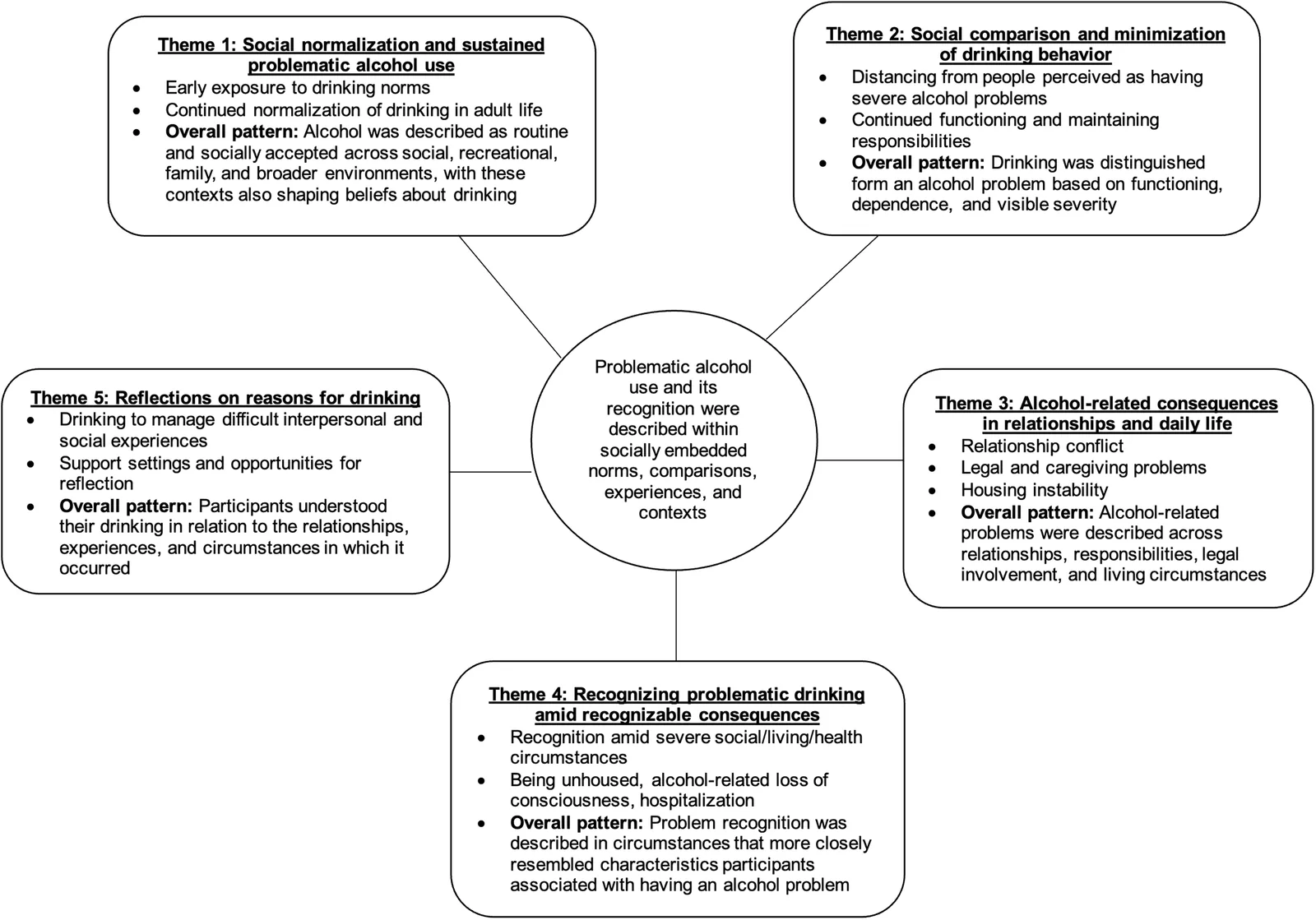
Thematic map depicting the social and contextual processes surrounding problematic alcohol use and its recognition.

#### 3.2.1 Social normalization and sustained problematic alcohol use

Participants described alcohol use as common and highly visible across different periods of their lives. Many recalled early experiences in families, peer groups, and community settings where drinking was treated as ordinary. Others described how alcohol continued to be closely tied to socializing, recreation, and everyday routines in adulthood. Across these accounts, repeated exposure to drinking and messages about what constituted acceptable alcohol use contributed to the normalization and continued use of alcohol.

*Early exposure to drinking norms*. Several participants described growing up in settings where drinking, including problematic drinking, was treated as routine or socially acceptable. One participant recalled how norms around drinking differed when he was younger: *“When I was younger, society was a little different. I can remember people drank while they drove. That was considered normal”* (66-year-old man). Others described problematic alcohol use within their families as familiar and not necessarily viewed as concerning: *“When I was growing up, functional alcoholics were pretty normal in society. My father was an alcoholic. He didn’t think it was a problem”* (66-year-old man). Another participant described growing up in a setting where alcohol was closely intertwined with her mother’s work and social life: *“I grew up in a bar, literally. My mom worked in a bar the whole time that I was growing up. So that was my mom’s community. And so, she had a job where you could drink while you were there. I have all these pictures of me as a little girl flocking around in the bar. Alcohol was very normalized”* (53-year-old woman). Peer settings also shaped some participants’ early experiences with alcohol, particularly when drinking was associated with fitting in and having fun. One participant explained: *“I was doing this to fit in. This is what I’m supposed to do to have a good time”* (55-year-old woman). Together, these accounts illustrate how some participants encountered alcohol from an early age in settings where drinking was familiar, socially accepted, and closely connected with family and social life.

*Continued normalization of drinking in adult social life*. Participants also described alcohol as remaining a routine part of social and recreational life in adulthood. One participant described alcohol as woven into social, recreational, and everyday activities: *“It’s just naturally a part of life to have drinks. We’re playing golf, and there’s going to be alcohol. We’re going to watch a game … there’s going to be alcohol. We’re just sitting, talking, or chilling…. there’s going to be alcohol”* (39-year-old man). This association between drinking and social life was also reflected in broader messages and representations of alcohol: *“If I went on Facebook and I found something on Saturday night, usually, it involved drinking”* (46-year-old woman). Another participant described both the availability of alcohol and its frequent promotion in advertising, particularly around sports: *“That definitely made it hard because we have vodka and brandy and stuff. So, if I wanted to drink, I could. But the thing that really made it hard on me was the amount of commercials, especially like sports and stuff like that, it’s in every ad [drinking]”* (45-year-old man).

Participants also described how familial messages about drinking contributed to the continued normalization of drinking and beliefs about acceptable alcohol use in adulthood. One participant described how a family member equated acceptable drinking with the ability to continue meeting daily responsibilities:“My uncle always used to say that if a man can drink, it doesn’t matter how F***ed up you get, as long as you can make it to work the next day, that’s all that counts. So, I kind of lived my life like that” (56-year-old man).

Participants also described drinking as closely tied to socializing and having fun. One participant stated that being around other people was associated with increased alcohol use: *“I don’t know if it was boredom or just the wanting to fit in part of it. But I did notice that I was drinking more when I was with people”* (55-year-old woman). Another participant similarly described his alcohol use as connected to having fun rather than drinking alone: *“My alcohol problem was more like, ‘I just love to have fun.’ It never was, ‘Let me go sit in the corner and get drunk’”* (56-year-old man). Across these accounts, alcohol remained embedded in adult life through broader social messages, family beliefs, and social and recreational settings where drinking was common and socially accepted.

#### 3.2.2 Social comparison and minimization of drinking behavior

Participants described evaluating their own alcohol use in relation to people they perceived as having more severe alcohol problems. These comparisons often centered on dependence, visible impairment, and the ability to maintain daily responsibilities. One participant described recognizing the negative effects from drinking while distinguishing her experience from what she considered alcohol dependence: *“I didn’t know that I struggled with it in the past. I had seen that when I had too much to drink, I didn’t feel well. It made it more difficult to do my responsibilities. From that standpoint, it was a struggle the next day. But where I didn’t have the issue was the physical and emotional dependence on it”* (44-year-old woman).

Participants also distinguished their own drinking from having an alcohol problem based on whether they were still able to function in their daily lives. One participant explained: *“If you’re a type of alcoholic that needed to have a drink to get started for the day, that would be a problem. I wasn’t that type. I would work all day, and then I would drink after work. It’s more functional”* (66-year-old man). Participants’ descriptions of people they considered to have alcohol problems included terms such as *“disheveled,” “disorganized,” “unprepared,” “sloppy,” “unhoused,”* and *“miserable,”* as well as having legal or interpersonal problems and difficulty maintaining responsibilities. Participants’ ability to maintain daily responsibilities often contributed to the perception that their drinking was not problematic. Continued functioning and distance from visible or severe representations of alcohol problems served as reference points through which participants interpreted their own drinking as nonproblematic.

#### 3.2.3 Alcohol-related consequences in relationships and daily life

Participants described increasing difficulties in their relationships and daily lives alongside their alcohol use, including interpersonal conflict, legal problems, caregiving disruptions, and housing instability. One participant described relationship and legal problems accumulating over time: *“I started having relationship problems, lots of fighting, legal problems, tickets. I didn’t take care of my tickets. They turned into warrants”* (55-year-old woman). Other participants described significant effects on family relationships and caregiving roles. One participant reflected: *“I did a lot of damage to myself and those I love. My alcohol abuse really affected my life, which I never wanted it to. But by the time I had my own family, I was already in too deep to realize what was happening”* (40-year-old man). Another described losing parental rights following repeated legal consequences related to drinking: *“I got my third DUI, and that was just the beginning. I then lost all parental rights to my son”* (46-year-old woman). Participants also described housing instability alongside problematic alcohol use: *“I was homeless. And I was hooking up with men left, right, and center, trying to find one of them to take care of me, even though they were addicts and couldn’t take care of themselves. And I kept going around and around through the shelters”* (53-year-old woman). Across accounts, participants described alcohol-related problems across multiple areas of their social and living environments, including relationships, family responsibilities, legal involvement, and housing stability.

#### 3.2.4 Recognizing problematic drinking amid recognizable consequence

Participants described recognizing their drinking as problematic when changes in their social circumstances, living environments, or health more closely resembled the severe and visible characteristics they associated with having an alcohol problem. One participant described problem recognition in the context of increasingly disrupted circumstances surrounding his drinking, including passing out in public and outdoor spaces and being unable to make it home: *“I fell into a rut. And I had drank for about a month, the most I ever drank in my life. I was passing out on the street. It was pretty degrading. That’s another reason why I quit. I was waking up inside bushes. I was not able to make it home”* (56-year-old man). Being unhoused and entering a shelter also marked a point of problem recognition for one participant: *“It wasn’t until I was homeless and in a shelter that I realized like, ‘hey, I’ve got a problem’”* (55-year-old woman). Hospitalization similarly provided a setting in which another participant recognized that her drinking had become problematic: *“Because going from that much alcohol to none, those withdrawals sucked … I was 88 pounds, and in the hospital, and that’s when, I guess, the light came on. It was like, ‘I have a problem, I have to figure this out’”* (46-year-old woman).

#### 3.2.5 Reflections on reasons for drinking

Participants reflected on reasons they believed they had used alcohol and the contexts in which they made sense of their drinking. While participant accounts varied, alcohol was frequently described as a way of managing difficult interpersonal and social experiences. Support settings and periods of reflection also provided spaces to consider the role alcohol had played in their lives.

Participants described using alcohol to manage difficult interpersonal and social experiences. One participant described using alcohol as a way of managing difficulties in her relationship with her husband: *“I started drinking. And it’s not really an excuse. It’s just how it happened. My husband at the time was a cop, and he just got more and more possessive of me and his son. And I found the more drinks I had, the better I could deal with him”* (46-year-old woman). Another participant described using alcohol to manage loneliness, shyness, and discomfort around other people: *“And so I looked to stuff outside of me to try and help what I felt. I felt lonely. I felt very ugly. I was painfully shy. And so, I used alcohol to kind of numb. It made it easier to be around people, not feel so self-conscious. So, it just very quickly became the way I made myself feel better”* (53-year-old woman). Rather than occurring in isolation, drinking was described in relation to the social circumstances participants were navigating.

Participants also described support settings and periods of reflection as contexts for understanding their drinking. One participant also described Al-Anon as a setting where she reflected on family dynamics and developed a way of understanding experiences she connected with drinking: *“I started to work on myself when I was in Al-Anon because I started to understand a little bit of the family dynamics codependency meant, because codependency is the root of all addictions. And I’ve learned this. I’ve learned this. It makes sense to me. It’s like, ‘Oh, okay. That’s why that happens. That’s why people do these things.’ So, I started to work on myself’”* (62-year-old woman). Her account highlights how a support-group setting provided one context for reflecting on family relationships and drinking-related experiences. Another participant described recognizing alcohol as problematic when she had greater periods of reflection to consider its role in her life: *“For me, I had the kind of time and mental space to identify that alcohol was a disruptor. It’s not something I need any more to escape some bogeyman that doesn’t exist in my life”* (44-year-old woman). In these reflections, support settings and circumstances that afforded time and mental space were described as contexts for understanding alcohol use.

## 4 Discussion

This study explored how individuals in recovery from AUD described the socioenvironmental contexts surrounding the normalization, sustained use, and recognition of problematic alcohol use. Findings illustrate how socioenvironmental contexts normalized alcohol use and informed participants’ perceptions of what constituted problematic drinking. Problem recognition was described when participants’ experiences became more closely aligned with these contextually informed characteristics of problematic drinking. These findings suggest that socioenvironmental contexts may influence not only alcohol use, but also how individuals interpret whether their own drinking constitutes a problem.

Consistent with SCT and social-ecological frameworks (12, 54, 55), findings suggest that unhealthy alcohol use is embedded within the social and environmental contexts where individuals live and interact. The ubiquity of alcohol use across societal, family, and peer environments created strong normative influences and repeated opportunities for observational learning. Within these contexts, problematic alcohol use was often perceived as socially acceptable or routine rather than problematic. These findings are consistent with prior literature demonstrating that the intersection of societal, social, and cultural norms contribute to the normalization and escalation of alcohol use (56). They also align with evidence showing that environmental factors, including early household substance use exposure, account for a substantial portion of AUD risk, often exceeding the variance explained by genetics alone (57). Overall, problematic alcohol use may be under-recognized when it is primarily indexed through more visible, recognizable markers such as solitary drinking (58).

Participants often distinguished their own drinking from an alcohol problem when they could still work, meet responsibilities, and/or did not view themselves as dependent. Even when negative effects from drinking were present (e.g., struggling the next day due to heavy drinking the day prior), continued functioning remained an important distinction between their drinking and what they considered problematic. Prior research suggests that individuals who maintain daily responsibilities and social roles, and who do not conform to stereotypical images of problematic alcohol use, may be less likely to identify their drinking as concerning (59). The continued ability to fulfill expected social and daily roles may reinforce perceptions that drinking is not problematic, even as alcohol-related consequences emerge.

Participants also contrasted themselves with people they associated with more severe and recognizable alcohol-related problems. Qualitative studies have similarly found that individuals may distinguish and distance their own drinking from more severe or stereotypical images of problematic drinking (e.g., dependence, loss of control, impaired functioning), while describing their own as controlled or responsible (29, 60). Prior research has demonstrated that individuals with AUD are often stereotyped as dangerous, unpredictable, untrustworthy, and lacking control or moral character, which contributes to widespread societal stigma and discrimination (61, 62). These representations may be reinforced by a binary distinction between problematic (e.g., visible dysfunction) and non-problematic drinking (e.g., maintaining functional roles), which is based on highly visible markers of impairment and consequences (23). These distinctions may contribute to problematic alcohol use remaining under-recognized and may delay problem recognition among individuals who do not resemble severe and visible representations of AUD (23).

The present findings extend prior work by suggesting that socioenvironmental contexts informed not only what participants regarded as normal or acceptable drinking, but also the standards they used to recognize an alcohol problem in themselves. Messages from family that drinking remained acceptable as long as responsibilities were met, together with characteristics participants associated with more severe alcohol problems (e.g., being “unhoused,” “disorganized,” or “disheveled”), provided standards against which participants interpreted their own drinking. Problem recognition was described when participants’ circumstances more closely resembled these characteristics; for example, participants described experiencing housing instability, publicly visible or unsafe circumstances, and major relationship disruptions, including loss of child custody. Severe alcohol-related consequences and “hitting bottom” are well documented in the literature on problem recognition and decisions to change drinking (28, 63). Importantly, experiencing severe consequences does not necessarily result in recognizing one’s drinking as problematic or deciding to change. Not all individuals who experience severe alcohol-related consequences recognize their drinking as problematic or decide to change it. The present findings add to this literature by showing that participants described problem recognition in circumstances that resembled the severe and recognizable characteristics they associated with having an alcohol problem. This suggests that socially and environmentally informed understandings of problematic drinking may be relevant to the circumstances in which alcohol-related consequences are recognized as problematic.

Participant accounts also included reflections on reasons they believed they drank, including managing emotional distress and difficult interpersonal and social experiences. These findings align with literature demonstrating that individuals drink to cope with stress and negative affective states (64, 65). The present findings further situate these reasons for drinking within participants’ relationships and social circumstances, including difficult partnerships, loneliness, social discomfort, and distress associated with concealing important aspects of oneself. Participants also described support settings and circumstances that afforded time and mental space as contexts for reflecting on the role alcohol had played in their lives. Together, these accounts further illustrate how participants understood their drinking in relation to the social relationships, experiences, and environments in which it occurred.

### 4.1 Limitations and future directions

The study findings are considered alongside limitations. First, while this study provides insight into how individuals in recovery describe the process through which their unhealthy alcohol use developed, progressed, and was recognized, the data represent a single point in time, which captures participants’ current narrative of their past; importantly, this may differ from the perspectives that participants held while actively using alcohol. Future research may consider interviewing individuals who continue to actively use and are not in treatment to better understand how alcohol-related problems are interpreted or recognized prior to engagement in recovery-oriented spaces. Second, although the sample size (N = 15) is appropriate for an in-depth qualitative design and allowed for rich, detailed accounts, findings are not intended to be generalizable. Future research may examine whether similar patterns of AUD development and recognition emerge utilizing larger and more diverse samples that also capture different stages of alcohol use. Third, the AUDIT was completed retrospectively based on participants’ period of heaviest alcohol use. Although the ≥7 cutoff has been used in treatment-seeking populations, it has not been validated for retrospective use. Fourth, the sample spanned a wide age range, and participants may therefore have encountered different social, cultural, and historical contexts surrounding alcohol use across the life course. The study was not designed to examine age-related differences in these experiences. Fifth, although the inductive analytic approach was well positioned to identify patterns in participants’ narratives, the interview protocol was not originally designed to examine the development of alcohol use trajectories in depth. As a result, opportunities for targeted probing around the developmental patterns of alcohol use and recognition were limited. Future studies may build on these findings by creating interview protocols specifically designed to capture AUD development, escalating consequences, and the evolution of problem recognition. Sixth, the interview protocol did not specifically assess broader environmental determinants of alcohol use, such as alcohol outlet density, retail accessibility, affordability, or neighborhood socioeconomic conditions. Similarly, although current income and self-reported childhood and current social class were collected as demographic characteristics, the interview protocol did not inquire about participants’ socioeconomic or material circumstances during the period of problematic alcohol use. Accordingly, we were unable to examine how factors such as employment security, financial resources, or housing stability may have shaped the normalization or recognition of problematic alcohol use. Future research may examine how these structural, commercial, and socioeconomic circumstances relate to the normalization, sustained use, and recognition of problematic alcohol use.

Future research may examine how these structural and commercial environments relate to the normalization, sustained use, and recognition of problematic alcohol use.

### 4.2 Conclusion

Findings from this qualitative study suggest that socioenvironmental contexts may be relevant not only to the normalization and sustained use of alcohol, but also to how individuals interpret whether their own drinking constitutes a problem. Problem recognition was described when participants’ experiences more closely resembled the contextually informed characteristics they associated with problematic drinking. These findings have important implications for prevention and early identification. Current approaches to identifying problematic alcohol use often emphasize quantity and frequency of drinking, impaired control, consequences, and concerns expressed by others. Prevention and early intervention efforts may benefit from shifting away from crisis-driven models of care and towards an earlier recognition of unhealthy alcohol use within healthcare and community settings. Screening and brief intervention efforts may benefit from exploring how individuals themselves define problematic drinking and the social comparisons they use to evaluate their own alcohol use. Clinicians may need to emphasize that problematic alcohol use can occur even when individuals continue to work, maintain relationships, and fulfill daily responsibilities.

Public health messaging may similarly benefit from challenging binary and stigmatizing representations of AUD that associate alcohol problems primarily with severe or highly visible dysfunction. Increasing awareness that problematic alcohol use can coexist with continued functioning may help individuals recognize concerns before severe consequences or major life disruptions occur. Prevention efforts may also benefit from incorporating family-and systems-informed approaches that engage close social networks in identifying and responding to unhealthy alcohol use (66). In conclusion, these findings extend socioenvironmental perspectives on alcohol use by suggesting that social and environmental contexts may influence not only drinking practices and norms, but also how individuals understand and recognize their own alcohol use as problematic. Earlier recognition may therefore require attention to the standards individuals use to evaluate their drinking, rather than relying primarily on severe or highly visible consequences as indicators of an alcohol problem.

## Data Availability

The datasets presented in this article are not readily available because of confidentiality regarding the involvement of a vulnerable population. Requests to access the datasets should be directed to Monique T. Cano, monique.cano@yale.edu.
